# Health insurance and quality of care: Comparing perceptions of quality between insured and uninsured patients in Ghana’s hospitals

**DOI:** 10.1186/s12939-016-0365-1

**Published:** 2016-05-12

**Authors:** Aaron A. Abuosi, Kwame Ameyaw Domfeh, Joshua Yindenaba Abor, Edward Nketiah-Amponsah

**Affiliations:** Department of Public Administration and Health Services Management, University of Ghana Business School, P.O Box LG 78, Legon, Ghana; Department of Finance, University of Ghana Business School, Legon, Ghana; Department of Economics, University of Ghana, Legon, Ghana

**Keywords:** Health insurance, Quality of care, Insured, Uninsured, Ghana

## Abstract

**Background:**

The introduction of health insurance in Ghana in 2003 has resulted in a tremendous increase in utilization of health services. However, concerns are being raised about the quality of patient care. Some of the concerns include long waiting times, verbal abuse of patients by health care providers, inadequate physical examination by doctors and discrimination of insured patients. The study compares perceptions of quality of care between insured and uninsured out-patients in selected hospitals in Ghana to determine whether there is any unequal treatment between insured and uninsured patients in terms of quality of care, as empirical and anecdotal evidence seem to suggest.

**Methods:**

A cross-sectional survey of 818 out-patients was conducted in 17 general hospitals from three regions of Ghana. These are the Upper East, Brong Ahafo and Central Regions. Convenience sampling was employed to select the patients in exit interviews. Descriptive statistics, including frequency distributions, means and standard deviations, were used to describe socio-economic and demographic characteristics of respondents. Factor analysis was used to determine distinct quality of care constructs; *t*-test statistic was used to test for differences in quality perceptions between the insured and uninsured patients; and regression analysis was used to test the association between health insurance and quality of care.

**Results:**

Overall, there was no significant difference in perceptions of quality between insured and uninsured patients. However, there was a significant difference between insured and uninsured patients in respect of financial access to care. The major quality of care concern affecting all patients was the problem of inadequate resources, especially lack of doctors, lack of drugs and other basic supplies and equipment to work with.

**Conclusions:**

It was concluded that generally, insured and uninsured patients are not treated unequally, contrary to prevailing anecdotal and empirical evidence. On the contrary, quality of care is a concern of both insured and uninsured patients.

## Background

Health care financing has passed through a chequered history in Ghana. Following Ghana’s independence in 1957, public health services were provided free of charge through tax revenue [1, 2]. However, by the 1980s, this system of financing had become unsustainable. This led to the introduction of user fees by government. Individuals, therefore, paid out of pocket (OOP) for health services. However, the period of the user fees was characterized by serious challenges, key among which was inequity in access to health care, especially for the poor [3]. This resulted in decreased utilization of health care services at public health facilities [2]. Around 1990, the government of Ghana began to pilot a community-based health insurance schemes (CBHIS) as an option for financing health care. This culminated in the establishment of the National Health Insurance Scheme (NHIS) in October 2003 under Act 650 [4]. The primary aim of the NHIS is to improve access to and quality of basic health care services in Ghana through the establishment of mandatory district-level mutual health insurance schemes. It aims to replace OOP payments for health services and to provide financial protection against high costs of health care at the point of service [5]. Largely subsidized through government investment and value added taxes, Ghana’s NHIS asks for modest annual premium payments from its members, and many citizens are exempt from any payment at all [6]. The introduction of the NHIS, has led to a drastic increase in health service utilisation at all levels of health care in Ghana. According to the National Health Insurance Authority, general outpatient utilization of healthcare services increased over forty-fold from 0.6 million in 2005 to 25.5 million in 2011 [7]. It is reported that the total membership of the health insurance scheme constitute 33 % of the total population of Ghana [7].

Even though the increased utilization of health services is commendable, concerns are raised about quality of care in Ghana’s health care institutions. Some of the quality concerns include long waiting times for insured clients, verbal abuse of patients by health care providers, patients not being physically examined by doctor and unequal treatment given to insured and uninsured patients by health care providers [8–13]. These concerns, if genuine and persistent, have the potential to undermine the successful implementation of the nascent NHIS, since the success of any health insurance scheme partly depends on the quality of services beneficiaries of the scheme enjoy [14].

Most studies in developing countries have examined perceptions of quality of care from the perspective of patients in general, regardless of their insurance status [15–19], or only insured patients [12]. A few of the comparative studies did not place emphasis on the problem of unequal treatment between insured and uninsured patients [10, 13]. This study seeks to fill this gap by comparing perceptions of quality of care between insured and uninsured patients in order to establish whether there are any differences, and whether indeed insured and uninsured patients are treated unequally in Ghana’s health care institutions. A comprehensive array of quality of care dimensions is employed in the study. Specifically, the dimensions of quality include financial access to care, fairness aspects of care, adequacy of resources, effectiveness of treatment, interpersonal aspects of care and technical aspects of care. Differences in quality of care between insured and uninsured patients may inform health insurance reform, with the aim of ensuring appropriate health care utilization among subscribers and of expanding health insurance coverage to people who are currently not enrolled [20].

The rest of the paper is structured as follows: section 2 presents a brief review of literature on health insurance and quality of care; section 3 presents the methods; section 4 presents results of the study; section 5 presents discussions of the results; and section 6 presents conclusions and implications of the study.

## Literature review

There is paucity of literature linking health insurance and quality of care. A number of studies generally conclude that improving the quality of care is critical to realising the full benefit of health insurance schemes [21–23]. Available literature on health insurance and quality of care could be categorized into five. Some studies indicate that health insurance is positively associated with improved quality of care. Perez (D), Ang (A) and Vega (WA) [24] assessed the distribution of perceived quality of care among a national Latino population sample, and the role of insurance in different patient subgroups. Overall, those who were insured gave significantly higher ratings of excellent/good (81 % vs. 71 %) compared to those who were uninsured. A logistic regression analysis showed that insurance availability had an odds ratio of 1.47 (95 % CI, 1.22–1.76) net of socio-demographic characteristics in predicting perceived quality of care among Latinos. Nguyen (H), Rajkotia (Y) and Wang (H) [11] report that insured people in Ghana incur out of pocket payment for care from informal sources and also pay for out of pocket drugs and tests not covered by NHIS at health facilities. Nevertheless, they paid significantly less than the uninsured.

Other studies, however, suggest that health insurance tends to have a negative influence on quality of care. Dalinjong (PA) and Laar (AS) [13] studied perceptions and experiences of health care providers and clients in two districts of Ghana and reported that health providers had negative attitudes towards insured patients. Most of the insured experienced longer waiting times, more verbal abuse and other various forms of discrimination against them, compared with the uninsured patients. The insured attributed their experiences to the fact that they were not making immediate out of pocket payments for services, as providers preferred clients who would make instant payments for health care services. Bruce (K), Narh-Bana (S) and Agyepong (A) [8] found that long waiting times tended to affect insured clients more, compared with the uninsured.

A third category of literature, however, indicates that quality of care experiences may differ between insured and uninsured patients in the same health facility, depending on the nature of the service provided or the attitude of the service provider. In the Nouna District in Burkina Faso, objective quality of care evaluations by Robyn (PJ), Sauerborn (R) and Bärnighausen (T) [20] showed that providers were less likely to weigh, take the temperature, perform a physical examination, use a stethoscope, and inform patients about the diagnosis of their illness, when the patients were enrolled in the community-based insurance (CBI). In contrast, CBI enrolment was positively associated with overall patient satisfaction (aOR = 1.51, *p* = 0.014), controlling for potential socio-demographic characteristics such as patient socio-demographic status, illness symptoms, history of illness and characteristics of care received. The authors, however, found that there was no difference between the enrolled and non-enrolled respondents about the availability of medicines. This study seems to be the opposite to Jehu-Appiah (C), Aryeetey (G), Agyepong (I), Spaan € and Baltussen (R) [10] who found that both insured and uninsured households had positive perceptions with regards to the technical (objective) quality of care, but were negative about providers’ attitudes (interpersonal quality of care). The attitude of staff towards insured patients also differs, even in the same health facility. In India, a focus group discussion with staff at ASHWINI hospital found that whereas some patients complain that the nurses in the hospital reproach them for ‘being uninsured’, some of the staff rather considered the insured patients as a nuisance [25].

A fourth category of literature indicates that insurance status has no influence on quality of care received by patients. Bauchet (J), Dalal (A), Mayasudhakar (P), Morduch (J) and Radermacher (R) [26] conducted a study on a community micro-insurance scheme in India to assess whether insurance status improves healthcare quality. It was found that being insured is not significantly associated with receiving better-quality care, even when controlling for several patient and facility characteristics. In Ghana, a study conducted by the National Development Planning Commission (NDPC) reports that less than 50 % of respondents indicated that quality has improved following the implementation of the national health insurance scheme [27].

Finally, several studies show that even though poor quality of care may affect both insured and uninsured patients in health facilities, it poses as a disincentive to enrolment or renewal of membership of health insurance scheme [14, 28–30].

## Methods

### Study setting

The study took place in three regions of Ghana, the Upper, Brong Ahafo and Central regions, representing the savanna, forest and coastal ecological belts of Ghana respectively. The Upper East Region of Ghana is located in the northeastern corner of Ghana. The region’s economy is based on agriculture, primarily cattle and cereals like millet, sorghum and rice. The region has a total of 174 healthcare facilities [31]. The Brong Ahafo Region lies in the forest zone and is a major cocoa and timber producing area. The northern part of the region lies in the savannah zone and is a major grain- and tuber-producing region. The region has a total of 200 healthcare facilities [31]. The Central Region is located in the coastal belt of Ghana. The major economic activities are agriculture and fishing. Small-scale manufacturing also takes place in food-processing, ceramic wares, as well as salt and soap industries. The region has a total of 158 healthcare facilities [31].

### Study design and data collection

We conducted a cross-sectional survey of patients seeking outpatient consultations in 17 general hospitals from three regions of Ghana. The three Regions were selected to represent the three ecological belts in Ghana. The Upper East Region represents the northern Savanna belt, the Brong Ahafo Region represents the middle forest belt and the Central Region represents the southern coastal belt. These ecological belts have peculiar socio-economic characteristics. To make the sample representative of the whole country, therefore, it was important to include the major ecological belts of the country. We selected six hospitals each from the Brong Ahafo and Central Regions, consisting of two each of government, private and faith-based hospitals. However, in the Upper East Region there was only one faith-based hospital, therefore five hospitals were selected.

A body of literature indicates that perceptions of quality of care often vary among patients in government, private-for-profit and faith-based health facilities [32–36]. It was therefore necessary to select respondents according to types of health care providers. Both insured and uninsured patients were selected for interview. Fifty patients per facility were planned for interviews and we were able to obtain responses from 818 completed interviews. Over the study period, we employed convenience sampling to select patients for exit interviews among all the patients seeking general outpatient services . In each facility, patients on routine visits to the hospital were contacted and those who consented were interviewed. Convenience sampling was considered appropriate because patients visited the health facilities and exited at different periods, and it was only those who consented who were interviewed. On average, approximately 13–15 patients were interviewed per day. In-patients were not included in the sample. It was generally difficult to find uninsured patients, as about 1 in 8 outpatients were uninsured. A quota of 40 % was therefore provided for the uninsured patients. In spite of this, in some hospitals it was not possible to obtain the 40 % of uninsured patients within the period of the data collection. In the final analysis, out of the 818 respondents, 66.5 % were insured, while 33.5 % were uninsured. From July 22nd to August 20th, 2013, we collected data by conducting exit interviews with patients after they had completed their visit and departed from the facility grounds. If a patient was less than 18 years of age, the adult accompanying that person participated in the interview. Interviews were conducted by field workers recruited and trained by the researcher. An instrument for assessment of quality health care was adapted from Haddad (S), Fournier (P), Machouf (N) and Yatara (F) [18, 19]. This scale has been successfully used in other developing nations, Burkina Faso, rural Vietnam and India, by other researchers [17, 20, 37, 38].

### Data analysis

A factor analysis was used to determine distinct quality of care constructs; *t*-test statistic was used to test for differences in quality perceptions between the insured and uninsured patients, and then a regression analysis was used to test the influence of health insurance status of patients on perceived quality of care. Prior to conducting the factor analysis, the Kaiser-Meyer-Olkin (KMO) measure of sampling adequacy and the Bartlett’s test of sphericity were performed. The KMO score generated was 0.74, exceeding the recommended value of 0.6 [39, 40] and Bartlett’s test of sphericity [41] was highly significant (Chi-square 4437.615, df 276, *p* < 0.001), and thus supported the appropriateness of using the factor analysis to explore the underlying structure of the perceived quality of healthcare services.

An ordinary least squares (OLS) regression analysis was done to test the association between health insurance and quality of care. The dependent variable was the perceived quality of care which was a continuous variable. Insurance was a dummy, and coded 1 = insured; and 0 = uninsured (reference).

The rest of the independent variables were control variables. They included age of respondent, a continuous variable, ranging from 17 to 92 years; sex of respondent, a dummy variable coded, 1 = male, 0 = female (reference); marital status, a dummy, coded 1 = married, 0 = not married (reference); Education was a categorical variable, coded 1 = none (reference); 2 = primary; 3 = JHS; 4 = secondary/technical/vocational; 5 = tertiary. Income (monthly earnings) was a categorical variable, coded 1 = no earnings (reference); 2 = $10 or below; 3 = $11–25; 4 = $26–50; 5 = $51–100; 6 = $101–150; 7 = $151–200; 8 = $201–250; 9 = $251 and above. Distance to hospital was a continuous variable, ranging from 1 to 58 Km; Health status was measured using a Likert type scale ranging from 1, very poor, to 5 very good health status; Household size was a continuous variable. Hospital size was a continuous variable, and was measured by bed capacity (number of beds) as a proxy, ranging from 16 to 331 beds; Number of doctors was a continuous variable, ranging from 1 to 6 doctors per hospital; Region was a categorical variable, and coded 1 = Upper East (reference); 2 = Brong-Ahafo Region; 3 = Central Region. Ownership was a categorical variable, and coded 1 = government hospitals (reference); 2 = mission hospitals; 3 = private hospitals.

## Results

### Descriptive statistics

Tables 1 and 2 present the socio-economic and demographic characteristics of respondents.

**Table 1:** Socio-economic and demographic characteristics of all patients

Characteristics	N	Frequency		Mean
		No.	%	
Sex	817			
Male		334	40.6	
Female		484	59.4	
Marital status	810			
Unmarried		258	31.9	
Married		552	68.1	
Educational	817			
None		193	23.6	
Primary-JHS		351	43	
Sec/Tech/Vocational		153	18.7	
Tertiary		120	14.7	
Income	805			
GH¢100 and below		211	26.2	
GH¢ 101 and above		337	41.9	
No earnings		257	31.9	
Age	793			35.5
Household size	723			5.40

**Table 2:** Socio-economic and demographic characteristics of insured and uninsured patients

Characteristics	Insured			Uninsured		
	Frequency		Mean	Frequency		Mean
	No.	%		No.	%	
Sex						
Male	195	36		139	51	
Female	349	64		135	49	
Marital status						
Unmarried	151	28		107	39	
Married	389	72		163	61	
Educational						
None	121	22		72	26	
Primary-JHS	242	45		109	40	
Sec/Tech/Vocational	100	18		53	19	
Tertiary	80	15		40	15	
Income						
GH¢100 and below	226	42		98	36	
GH¢ 101 and above	154	28		82	30	
No earnings	163	30		94	34	
Age			35			37
Household size			5.25			5.7

From Table 1, out of the 817 respondents, a greater proportion (68 %) were females. With respect to marital status, a higher proportion of the respondents were married, while the least proportion (31.9 %) were unmarried. The results further showed that close to one-fourth: 193(24 %) had no formal education, 120 (15 %) had tertiary level education. A higher proportion: 239 (29 %) had middle/JHS education. The mean age of respondents was 36 years respectively. The mean household size of respondents was 5.4.

Table 2 shows that whereas the proportion of insured females were higher (64 %) compared with insured males (36 %), the proportions were almost the same in the case of uninsured females and males (49 % and 51 % respectively). The results also indicate that among those married, the insured were more (72 %) compared with the uninsured (61 %), whilst those who were not married 28 and 39 % respectively. With respect to educational background, there were no significant differences between the insured and uninsured regarding the proportions of respondents who completed the various levels of education. Twenty-two percentage (22 %) of the insured and 26 % of the uninsured had no formal education; 45 % of the insured and 40 % of the uninsured had primary/junior high school level education; 18 % of the insured and 19 % of the uninsured had secondary/vocational/technical level education; and 15 % respectively of the insured and uninsured had tertiary level education. Among those who earned monthly incomes of GHC100 ($50) or below, the insured earned a relatively higher proportion (42 %) compared with the uninsured (36 %). However, among those who earned GHC101 ($51) or above, both insured and uninsured earned similar proportions of 28 and 30 % respectively. The proportions were also similar with those who received no monthly earnings (30 and 34 % respectively). There were also no marked variations in the ages and household sizes of insured and uninsured respondents. The mean age of the insured was 35, whereas that of the uninsured was 37. On the other hand, the mean household size of the insured was 5.25; whereas those of the uninsured was 5.7.

### Factor analysis of the quality of care

Table 3 presents results of the factor analysis.

**Table 3:** Factor analysis of the quality of care scale

Rotated component matrix^a^						
	Component					
	1	2	3	4	5	6
Financial access						
NHIS pays the cost of all treatment	.721					
Cost of services are affordable	.711					
Exempted patients are treated free of charge	.708					
Only official fees are charged	.570					
Fairness of care						
Staff treat all patients fairly		.764				
Quality of drugs is same for all patients		.763				
Patients are treated on first-come-first-served basis		.620				
Very ill patients are treated first		.586				
Adequacy of resources & services						
Doctors are sufficient			.784			
Supplies are sufficient			.739			
Rooms in OPD are sufficient			.673			
Waiting time is reasonable			.651			
Drugs are available			.473			
Effectiveness of treatment						
Pharmacy instructions are clear				.585		
Treatment is effective for recovery and cure				.501		
Quality drugs are given to patients				.445		
Technical care						
Patients are told diagnosis					.738	
Patients are physically examined					.615	
Lab. and other tests are done					.610	
Patients are involved in their care					.557	
Interpersonal care						
Staff show compassion & support to patients						.865
Staff are polite & respectful to patients						.861

Extraction Method: Principal Component Analysis

Rotation Method: Varimax with Kaiser Normalization

a. Rotation converged in 6 iterations

The initial scale used in data collection had 28 items divided into five main dimensions. However, the factor analysis reduced the items to 22, divided into six dimensions, namely, financial access to care, fairness aspects of care, adequacy of resources, effectiveness of treatment for recovery and cure, technical aspects of care, and interpersonal aspects of care.

The scale was tested for reliability. It had an overall Cronbach’s alpha value of 0.79, while the subscales ranged from 0.58 to 0.84. Thus, the reliability was highest for ‘interpersonal aspects of care’ (0.84) and lowest for ‘technical aspects of care’ (0.58). The overall mean score was 89.11, while the standard deviation was 11.457. Respondents could express their perceived quality of care on a five-point Likert scale: strongly disagree (1), disagree (2), neutral (3), agree (4), strongly agree (5).

### Assessment of levels of quality of care

For purposes of assessing the levels of perceived quality of care, the mean ratings of the various indicators under a dimension was added to obtain a total quality care index for that dimension, for both the insured and uninsured patients. In the case of financial access, for instance, there are four indicators of quality of care. Based on the 5-point Likert scale, if all ratings of the indicators for this dimension were to be 1 (strongly disagree), then the total rating for financial access would be 4 (i.e. 1 × 4). If all ratings were 5, then the total rating would be 20 (i.e., 5 × 4). For ease of interpretation, the study also considered mean aggregate ratings between 4 and 12 as unfavourable or low quality; ratings between 12.01 and 16 as fairly favourable or average quality ratings; and ratings between 16.01 and 20 as favourable or good quality rating. The same approach was followed in determining the overall perception of quality of care, where ratings between 24 and 72 were considered unfavourable, 72.01 to 96, fairly favourable and 96.01 to 120, favourable.

### Perceptions of quality of care between insured and uninsured

An independent sample *t*-test was used to compare differences in perceptions of quality of care between the insured and uninsured. Table 4 shows the results of the comparison.

**Table 4:** T-test on perceived differences in quality of care between insured and uninsured patients

Indicators of quality of care	Insured (*N* = 544)		Uninsured (*N* = 274)		t-test
	Mean	Std. Dev.	Mean	Std. Dev.	
Financial access					
NHIS pays the cost of all treatment	3.85	1.16	3.48	1.16	4.073***
Cost of services are affordable	3.52	1.11	3.20	1.24	3.522***
Exempted patients are treated free of charge	3.97	1.06	3.52	1.09	5.396***
Only official fees are charged	3.70	1.15	3.89	1.04	−2.376**
Fairness of care					
Staff treat all patients fairly	3.40	1.36	3.49	1.32	0.863
Quality of drugs is same for all patients	3.37	1.30	3.40	1.30	0.316
Patients treated on first-come-first-served basis	3.92	1.34	3.97	1.28	0.487
Very ill patients are treated first	4.26	1.13	4.22	1.12	−0.457
Adequacy of resources & services					
Doctors are sufficient	2.79	1.30	2.92	1.28	−1.303
Supplies are sufficient	3.14	1.13	3.04	1.17	−1.136
Rooms in OPD are sufficient	3.24	1.31	3.24	1.29	0.080
Waiting time is reasonable	2.75	1.37	2.94	1.30	−1.897*
Drugs are available	3.29	1.27	3.33	1.23	0.354
Effectiveness of treatment					
Pharmacy instructions are clear	4.56	0.63	4.51	0.65	0.988
Treatment is effective for recovery and cure	4.14	0.86	3.99	0.85	2.349**
Quality drugs are given to patients	3.97	0.91	3.92	0.86	0.714
Technical care					
Patients are told diagnosis	3.38	1.48	3.30	1.44	0.691
Patients are physically examined	3.92	1.24	3.75	1.35	1.745*
Lab. and other tests are done	3.92	1.26	3.64	1.35	2.838**
Patients are involved in their care	3.68	1.32	3.71	1.22	−0.274
Interpersonal care					
Staff show compassion & support to patients	4.08	0.97	4.19	0.89	−1.519
Staff are polite & respectful to patients	4.12	0.98	4.19	0.91	−1.008
Overall perceived quality of care	89.48	11.263	88.38	11.836	−1.212

*p ≤ .10; **p ≤ .05; ***p ≤ .01 (two-tailed test)

The study reveals that overall, there is no significant difference in perception of quality of care between insured and uninsured patients (Insured: M = 89.48, SD = 11.263; Uninsured: M = 88.38, SD = 11.84) *p* = 0.226. Regarding the level of quality of care, both insured and uninsured respondents gave a fairly favourable rating, indicating that overall, quality of care in Ghana’s hospitals is somewhat good.

With respect to the individual indicators of quality of care however, there is a significant difference in perceptions of quality of care between the insured and uninsured in respect of all indicators of financial access to care (Insured: M = 3.85, SD = 1.16; Uninsured: M = 3.48, SD = 1.16) *p* < .001; costs of services being affordable (Insured: M = 3.52, SD = 1.11; Uninsured: M = 3.20, SD = 1.24) *p* < .001; and exempted patients treated free of charge (Insured: M = 3.97, SD = 1.06; Uninsured: M = 3.52, SD = 1.09) *p* < .001; and payment of unofficial fees (Insured: M = 3.70, SD = 1.15; Uninsured: M = 3.89, SD = 1.04) *p* < .018. In view of the special significance attached to financial access to health care, a multiple regression analysis was further performed to determine the association of respondents’ health insurance status on financial access to health care. The dependent variable was financial access, with insurance as the main independent variable, controlling for socio-demographic and hospital characteristics. The results indicate that insurance status is a significant predictor of financial access to health care (B = .805, *p* = .002). This suggests that for every additional person enrolled with the Ghana health insurance scheme, access to health care is increased by .805 points, holding other variables fixed. With regard to the levels of quality, both insured and uninsured respondents rated financial access to care fairly favourable (somewhat desirable) for all indicators.

Concerning respondent’s perceptions of fairness of care, there is no significant difference between the insured and uninsured in all indicators of quality. Three out of the four indicators of fairness of care were rated fairly favourably, while the indicator ‘Very ill treated first’, was rated favourably.

With respect to adequacy of resources, there is no significant difference in perceptions of quality between insured patients in all indicators except waiting time at 10 % significance level (Insured: M = 2.75, SD = 1.37; Uninsured: M = 2.94, SD = 1.30) *p* < 058. This implies that insured patients perceive waiting time to be longer, compared with uninsured patients. Two indicators, that is, adequacy of doctors and waiting time were rated unfavourably, and the remaining three indicators were rated fairly favourably.

On effectiveness of treatment, only the indicator ‘treatment is effective for recovery and cure’ shows a significant difference in perceptions of quality between insured and uninsured patients (Insured: M = 4.14, SD = 0.86; Uninsured: M = 3.99, SD = 0.85) *p* < 019. This suggests that more insured respondents considered the treatment they received from the hospital to be effective for recovery and cure. Regarding the levels of quality, two of the three indicators of effectiveness of treatment were rated favourably by respondents, while the indicator ‘Quality drugs are given to patients’ was rated fairly favourably by both insured and uninsured patients.

Regarding technical aspects of care, the indicator ‘laboratory and other tests are done’ shows a significant difference in perceptions of quality between insured and uninsured patients. The implication is that compared with uninsured patients, the treatment of insured patients is based more on confirmation of laboratory and other tests. At 10 % significant level, there was also a significant difference between insured and uninsured patients in respect of ‘Patients are physically examined’ (Insured: M = 3.92, SD = 1.24; Uninsured: M = 3.75, SD = 1.35) *p* < 082. On levels of quality of care, however, both insured and uninsured patients gave fairly favourable ratings of the technical aspects of care.

Finally, there is no significant difference between insured and uninsured patients regarding interpersonal aspects of care. However, both insured and uninsured respondents rated interpersonal aspects of care favourably.

### Association between health insurance and quality of care

Beyond evaluating differences in perceptions of quality of care between insured and uninsured patients, the study further used a multiple regression analysis to examine the association between health insurance and overall perception of quality of care, controlling for socio-demographic and hospital characteristics. Table 5 presents the results of the regression analysis.

**Table 5:** OLS multiple regression of the association of health insurance status on perceived quality of care

Independent variables	*b*	Beta
(Constant)	64.115	
Insurance status (Insured = 1)	1.065 (.927)	.044
Age of respondent	.067* (.031)	.083
Marital status (Married = 1)	.244 (.949)	.010
Sex (Male = 1)	−.922 (.923)	−.040
Educational level (None = 1)	−.306 (.292)	−.043
Income level (No earnings = 1)	−.330 (170)	−.076
Distance to hospital in Km.	.007 (.056)	.005
Health status	1.542** (.466)	−.215
Number of doctors	−1.699** (.493)	−.139
Size of household	.309 (.167)	.074
Bed capacity	.036*** (.008)	.287
Ownership of hospital (Government hospitals = 1)	3.401 (.595)	.247
Region (Upper East = 1)	4.102*** (.600)	.295

Dependent variable: Perceived quality of care. *p ≤ .10; **p ≤ .05; ***p ≤ .01

From Table 5, the results show that there is no significant relationship between insurance status and respondents’ perceptions of quality of care (B = 1.065, *p* = .251). In other words, the insurance status of patients in Ghanaian hospitals is not a predictor of perception of quality of care. For the control variables, however, there was a significant relationship between age of respondents and perceptions about the quality of care (B = .067, *p* = .033). The results show that an increase in respondents’ age by one year will result in improved perceptions of quality of care by .067 points, holding other variables fixed. This implies that as patients mature in age, they are more positive in their perceptions of quality of care. In order to confirm the linearity between the age of respondents and the dependent variable, age was squared in the regression model and analysed further. The results indicate that both age and age squared are not significant predictors of perceived quality of care. This suggests that age has a linear relationship with perceived quality of care. Other variables that have a positive and significant relationship with perceptions of quality of care include health status (*p* = .001), number of doctors (*p* = .001), hospital size (*p* = .001), ownership of hospitals (*p* < .001), and Region (*p* < .001).

## Discussion

The study finds that, overall there is no significant difference in perception of general quality of care between insured and uninsured patients and controlling for socio-demographic and hospital factors, that insurance status is not a predictor of quality of care in Ghana’s hospitals. This is inconsistent with some previous studies [20, 24]. However, individual indicators of quality of care show that there is a significant difference between insured and uninsured patients with respect to financial access to care. The insured had a more favourable view on all indicators of financial access. This is evident that the Ghana health insurance scheme has improved financial access to health care. This is consistent with previous studies [7, 10, 21]. Notwithstanding the improved financial access, the finding that some health care providers collect unofficial/informal fees in spite of the health insurance scheme, coupled with the fact that both insured and uninsured patients rate all indicators of financial access to care fairly favourably, implies that a great majority of patients still face problems as to financial access to health care.

There is no significant difference in perceptions of fairness of care between the insured and uninsured patients, this is contrary to anecdotal and empirical evidence that insured and uninsured patients are treated unequally, with the former receiving relatively poorer quality of care compared with the latter [11, 13].

There is no significant difference between the insured and uninsured patients regarding adequacy of resources and services, except on waiting time which is less favourable to insured patients. This finding is consistent with previous studies [8, 10, 12, 13]. Jehu-Appiah (C), Aryeetey (G), Agyepong (I), Spaan (E) and Baltussen (R) [10] found long waiting time as causing dissatisfaction among the insured patients in Ghana, and thus recommended an urgent action to be taken to address the problem. The unfavourable rating on adequacy of doctors is an important quality concern that must also be addressed in Ghana’s hospitals.

The strong positive ratings on pharmacy instructions on medicine intake is clear; and effectiveness of treatment for recovery and cure, is commendable. The significant difference between the insured and uninsured on effectiveness of treatment for recovery and cure could be due to the fact that the insured patients have better access to health care [7, 10, 21] and therefore are able to treat their illnesses early enough before they become complicated. It could also be due to the possibility of insured patients enjoying better health outcomes, resulting from accurate diagnosis based on laboratory and other diagnostic investigations. This conclusion is consistent with a study in Egypt [42] which found insured clients to have had a significantly higher frequency of physical examination and laboratory tests compared with their uninsured colleagues.

The favourable rating by both the insured and uninsured patients on interpersonal aspect of care finds support in literature. Juma (D) and Manongi (R) [34] assessed users’ perceptions of quality of care given at outpatient departments in Central Tanzania. Discussants stated that if patients are welcomed well with respect and compassion they assume that other services are good too.

## Conclusions

This study finds that overall, there is no significant difference in perceptions of quality of care between insured and uninsured patients. However, some indicators show significant differences in perceptions of quality of care, key among which is financial access to care. This is evidence that the implementation of the Ghana health insurance scheme has indeed improved financial access to health care. It is a good sign also that insured patients are more likely to undergo laboratory and other diagnostic investigations for accurate diagnosis of their illnesses, and are also more likely to report effectiveness of treatment for recovery and cure. These findings imply that every effort must be made to maintain the Ghana national health insurance scheme. The study thus concludes that generally, insured and uninsured patients are not treated unequally, in terms of quality of care in Ghana’s hospitals. On the contrary, insured patients have an advantage in terms of access to quality health care. The study however finds that generally, quality of care is fairly satisfactory to both insured and uninsured patients, which suggests that quality of care remains an issue deserving serious attention by health service providers. Critical quality issues which could have serious consequences for the successful implementation of the health insurance scheme include the collection of unofficial fees from patients by some healthcare providers, inadequacy of doctors and long waiting times. Thus, measures must be taken to address the unacceptable attitudes of some healthcare providers, as well as improve the general level of quality of care in hospitals.

## Limitation of the study

The study focused only on out-patients, excluding in-patients. Generalizations may therefore be done with caution, since in-patients’ perceptions of quality of care may differ markedly due to the different expectations of care. Notwithstanding this, we argue that the out-patient department (OPD) is the gateway to almost all of the hospital services. It is reported that globally, 80 % of patients are attended at OPD [34]. Thus, studies aimed at addressing issues of out-patients remain important. Also, the assessment of quality of care is based on perceptions, which are largely subjective. However, perceptions of quality have been increasingly accepted as valid and important measures of health care quality [43, 44]. It should also be pointed out that cross-sectional data are only snap-shots of events during the time of study, and therefore the material does not usually allow for establishing causal relationships. Hence, this study can only contribute with descriptive analyses of the statistical associations in question. Even though the three regions selected represent the respective zones of Ghana, considering that Ghana has ten regions in all, generalizations may have to be done with caution.

A further study is required to assess in-patients perceptions of quality of care within the context of the implementation of the Ghana health insurance scheme. A further study is also required to explore the nature and magnitude of informal charges in health facilities, as well as to explore innovative ways of increasing the supply of doctors and reducing waiting times in health facilities.
